# Seepage field characteristic and stability analysis of tailings dam under action of chemical solution

**DOI:** 10.1038/s41598-021-83671-6

**Published:** 2021-02-18

**Authors:** Guangjin Wang, Bin Hu, Sen Tian, Min Ai, Wenlian Liu, Xiangyun Kong

**Affiliations:** 1 School of Resources and Environmental Engineering, Wuhan University of Science and Technology, Wuhan, 430081 China; 2 Engineering Research Center for Green Comprehensive Utilization of Metal Ore Tailings Resources, Faculty of Land Resources Engineering, Kunming University of Science and Technology, Kunming, 650093 China; 3 State Key Laboratory of Coal Mine Disaster Dynamics and Control, School of Resources and Safety Engineering, Chongqing University, Chongqing, 400044 China; 4 China Nonferrous Metals Industry Kunming Survey and Design Research Institute Co. Ltd., Kunming, 650051 China; 5 State Key Laboratory of Geomechanics and Geotechnical Engineering, Institute of Rock and Soil Mechanics, Chinese Academy of Sciences, Wuhan, 430071 China; 6 Hubei Key Laboratory for Efficient Utilization and Agglomeration of Metallurgic Mineral Resources, Wuhan, 430081 Hubei China

**Keywords:** Mechanical engineering, Mineralogy, Sedimentology, Hydrology, Natural hazards, Solid Earth sciences, Physical chemistry

## Abstract

As one of the important influencing factors of tailings dam stability, seepage field distribution within the dam is often affected by the tailings mineral characteristics. While the alkalinity or acidity of reservoir water and long term immersion will partially change the physical and mechanical properties of tailings. This study carried out permeability tests of tailings under the action of chemical solution. On this basis, a three dimensional (3D) model was constructed to analyze the velocity field and effective saturation within the tailings dam. Moreover, the dam section along the valley bottom was selected as the basic section in calculation, so as to analyze the changes in infiltration point and buried depth of the phreatic line under different permeability coefficient ratios. The results suggest that, under the action of acid-alkaline solution, the permeability coefficients of tailings reduced, and the stronger solution acidity-alkalinity resulted in the longer action time and more obvious change; under the action of chemical solution, the fluid flow velocity in the dam gradually decreased, and the drat beach length in the reservoir gradually shortened. Besides, when the upper layer permeability coefficients of tailings was lower than that of the lower layer, the dam phreatic line had a shallow buried depth and a high infiltration point.

## Introduction

Tailings reservoirs are the essential solid waste storage facilities in mine production, which are also the kind of man-made source of danger with high potential energy and high collapsing force^1–3^. Once dam break accident occurs, it is extremely likely to cause secondary disasters such as mudslide, landslide, water and soil contamination, and further aggravate the accident hazard. These disasters would result in immeasurable damage to people’s life safety, property safety, and the ecological environment^4–6^. Researches find that, the occurrence of dam break accident is the consequence of the synthetic actions of multiple factors, including torrential rain, overtopping, earthquake liquefaction, seepage failure, dam base instability, dam structural failure and increased load^7,8^. To explore its ultimate cause, dam instability mainly refers to the changes in tailings dam stress field and seepage field induced by the above-mentioned factors. Meanwhile, the tailings dam composition and the material physicochemical properties also directly affect the changes in seepage field and stress field^9,10^.

Tailings are the main damming materials, and the physicochemical properties of tailings mainly include grain composition, porosity, water content and degree of consolidation, which have determined the dam layering, permeability and compressibility^11,12^. Meanwhile, the tailings properties are not always unchanged during a long-term storage life. They are related to not only the mineral processing technology and means, but also the tailings chemical composition and hydrochemistry environment during the storage life^13,14^. Under the seepage action, several phenomena can be observed in the tailings, including flowing soil, contact flowing soil, piping, and contact scouring. When these deformations develop to a certain degree, they will threaten the stability of the whole dam; in some severe cases, they may lead to dam break and collapse^15^. The existing relevant studies on tailings reservoir stability mainly focus on the special working conditions, such as earthquake liquefaction, rainfall and overtopping, as well as the influences of seepage field and geostatic stress under conventional working conditions on the dam stability^16–18^. However, during the mining-mineral process, there are residual heavy metals and chemical reagents in the tailings waste residues after the grinding and fine crushing and flotation processes, which have changed the hydrochemistry environment in the reservoir^19^. Under such circumstances, the tailings is always immersed in the acid and alkaline reservoir water, while the long-term immersion and chemical reaction have partially changed the tailings particle size, pore size, mineral composition and surface morphology^20,21^. Therefore, the permeability and mechanical property of the entire dam are changed to some extent.

The consolidation and the chemiosmosis-induced cementation of tailings result in the heterogeneous tailings sedimentary soils at the vertical direction, its effective stress showed nonlinear distribution, and the coefficients of permeability were different in different layers^22–24^. On this basis, this study carried out varying-head permeability tests on tailings under different conditions to investigate the associations of tailings macroscopic mechanical characteristics with changes in microscopic components by laboratory tests and microscopic observations. Thereafter, the obtained results were adopted in combination with numerical simulation to investigate the dam stability affected by the chemical field, and to analyze the effects of acid and alkaline environments on the permeability characteristics of tailings sedimentary soil, as well as the variation of permeability coefficient of tailings materials.

## Experimental and computational method

In this study, the research object was located in Sichuan Province of China, which was an upstream valley tailings reservoir, with the final elevation of 2090 m (the height above sea level), the total dam height of 147 m. The overall dam storage capacity is approximately 38.4359 million *m*^3^, the effective storage capacity is approximately 33.8928 million *m*^3^, and the fill dam height is 115 m*.* The sub-dams (23 steps) are built upstream from the main dam, so as to increase the storage capacity of the tailings reservoir. The initial dam of that tailings reservoir was the compacted rockfill seepage dam, with the dam height of 32.0 m.

### Experimental process

In this study, the TST-55 quintuplet varying-head permeability device was adopted for permeability tests. At the same time, water tank, beaker with scale, measuring cylinder, height-adjustable iron support and thermometer were also utilized to measure the coefficient of permeability of tailings materials.The tailings material permeability tests were performed according to the permeability test module in the *Standard for Soil Test Method* (GT/B 50123-2019)^25^, and sample loading and tests were carried out following related stipulations.

Representative tailings sandy silt, tailings floury silt and tailings silty clay were selected, which were immersed with the H_2_SO_4_ solution at pH = 3 and pH = 5 and NaCl solution at pH = 9 and pH = 12 for 120 days, respectively. Samples were collected for every 15 days for permeability tests.

### Model and computational parameters

**Figure 1:**
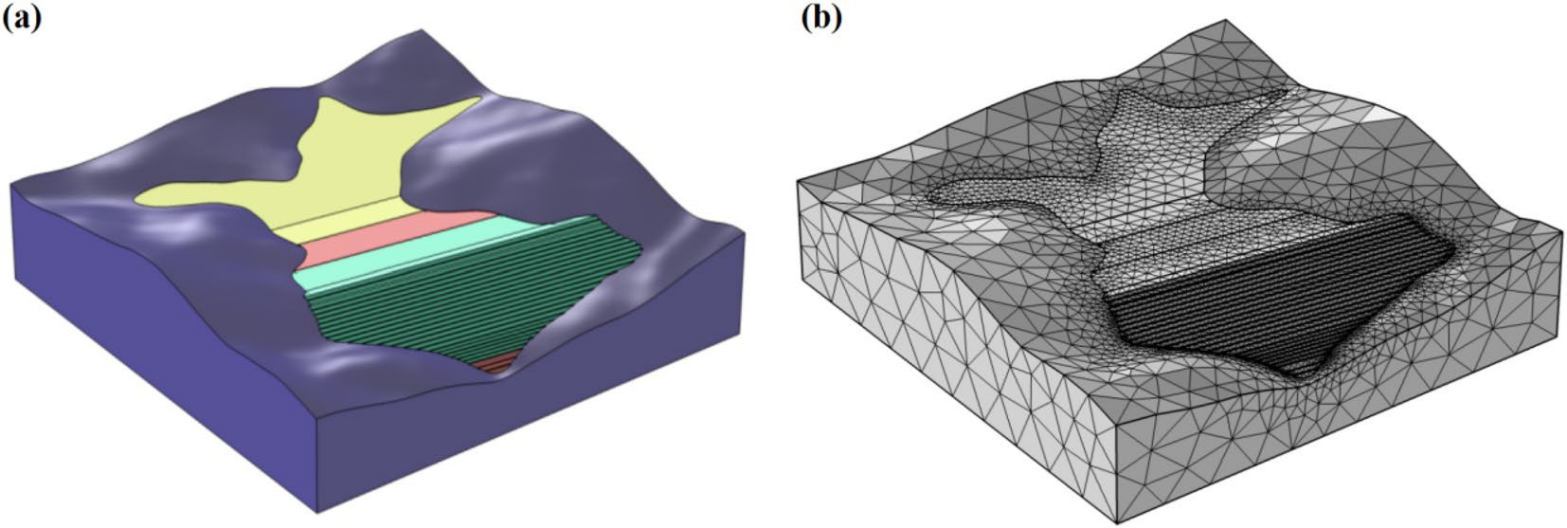
The model of tailings reservoir. (**a**) The 3D computation model of tailings reservoir and surrounding surface; (**b**) The geometric model and the grid partition.

The COMSOL Multiphysics is a kind of large finite element software, which is mainly used for scientific calculation and simulation in diverse engineering fields^26–28^. Based on this software, the 3D model of this tailings reservoir and the surrounding surface was constructed (Fig. 1), and the V-G model in the Richards equation was selected to calculate the seepage field of this tailings dam. At the same time, the model was subjected to grid partition, as shown in Fig. 1b, the model was divided into 5 computed fields according to the actual engineering, among which, the bed rock part was not considered in computation. The calculation parameters of the other 4 computed fields are shown in Table 1.

**Table 1:** The basic calculation parameters of the V-G model.

Fields	$\theta_{S}$	$\theta_{r}$	$\alpha$(1/m)	n	$K_{S}$(cm/s)
Initial dam	0.2857	0.0229	2.94	1.8089	5e-3
Tailing silty sand	0.4012	0.0335	3.21	2.0066	–
Tailing silty soil	0.3976	0.0371	3.44	2.3155	–
Tailing silty clay	0.3865	0.0399	3.67	2.5267	–

**Figure 2:**
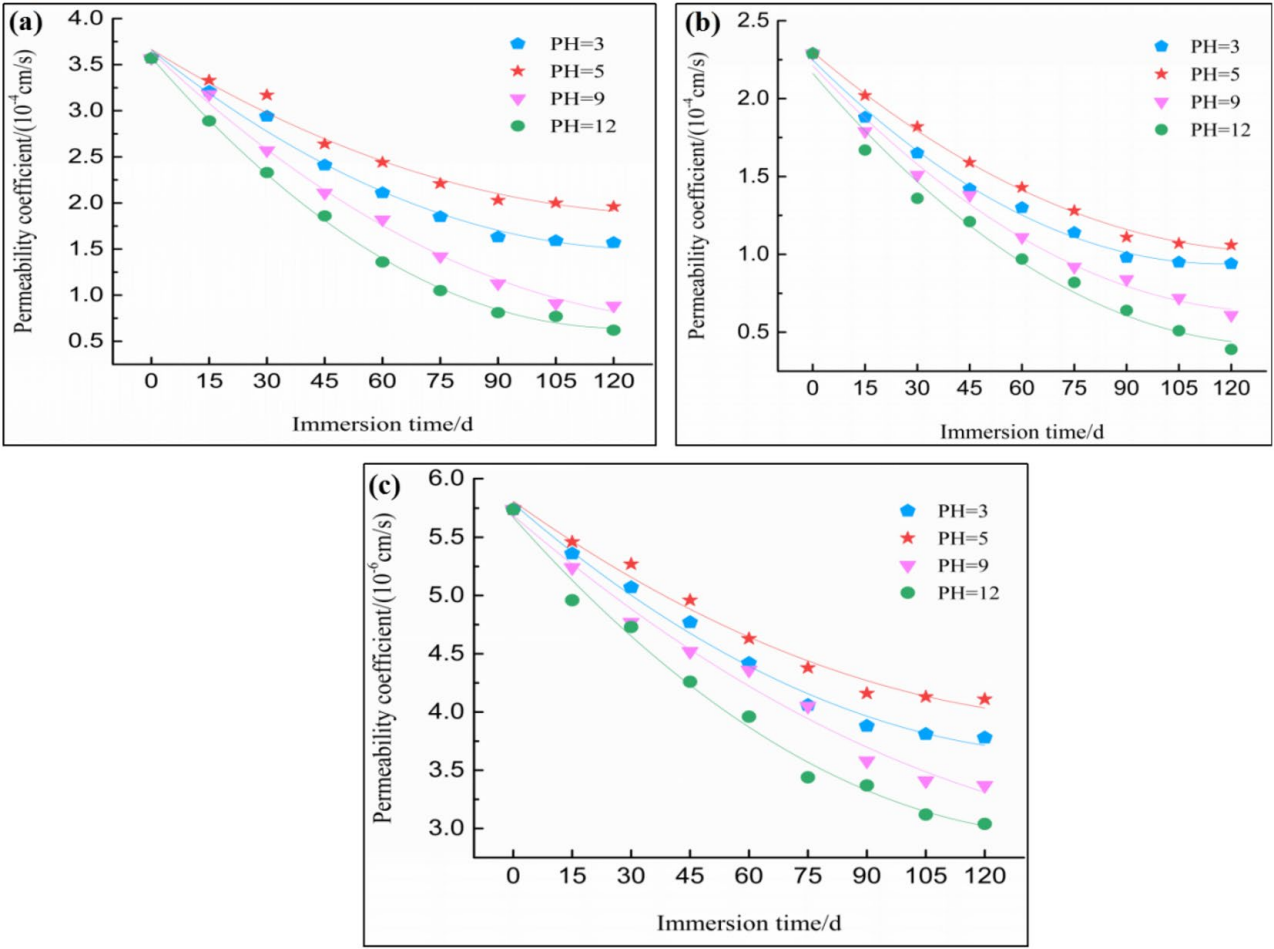
The relationship between the permeability coefficient of tailings and immersion duration. (**a**) Tailing silty sand; (**b**) Tailing silty soil; (**c**) Tailing silty clay.

In addition, when the fill dam height of the tailings dam was determined, the buried depth of the phreatic line was mainly affected by the saturated permeability coefficient ($K_{S}$) of each layer in the dam^29–31^. Therefore, based on Fig. 2b, the effects of permeability coefficient changes of tailings materials after different chemical solution treatments on the dam velocity field and effective saturation field were investigated, with $K_{S}$ as the unique variable. The model parameters are displayed in Table 2.

**Table 2:** Permeability coefficient of various tailings materials under different chemical conditions.

Conditions	Damming materials		Permeability coefficient (cm/s)
1	Original sample	Tailing silty sand	3.57·10^–4^
		Tailing silty soil	2.29·10^–4^
		Tailing silty clay	5.74·10^–6^
2	After chemical solution (pH = 3) treatment for 120 days	Tailing silty sand	1.57·10^–4^
		Tailing silty soil	0.94·10^–4^
		Tailing silty clay	3.78·10^–6^
3	After chemical solution (pH = 5) treatment for 120 days	Tailing silty sand	1.96·10^–4^
		Tailing silty soil	1.06·10^–4^
		Tailing silty clay	4.11·10^–6^
4	After chemical solution (pH = 9) treatment for 120 days	Tailing silty sand	0.886·10^–4^
		Tailing silty soil	0.61·10^–4^
		Tailing silty clay	3.37·10^–6^
5	After chemical solution (pH = 12) treatment for 120 days	Tailing silty sand	0.62·10^–4^
		Tailing silty soil	0.39·10^–4^
		Tailing silty clay	3.04·10^–6^

## Results and discussion

### Analysis of experimental result

Permeability tests were performed according to the above-mentioned testing program. Then, the relationships of immersion time and solution pH value with the permeability coefficient were constructed according to the obtained test data and results, as shown in Fig. 2. The results suggested that, chemical solution treatments, regardless of the acidity or alkalinity, reduced the permeability coefficient of tailings material, which displayed a relation similar to polynomial with the immersion time.

### Effect of tailings permeability coefficient on seepage field

#### Analysis of velocity field

**Figure 3:**
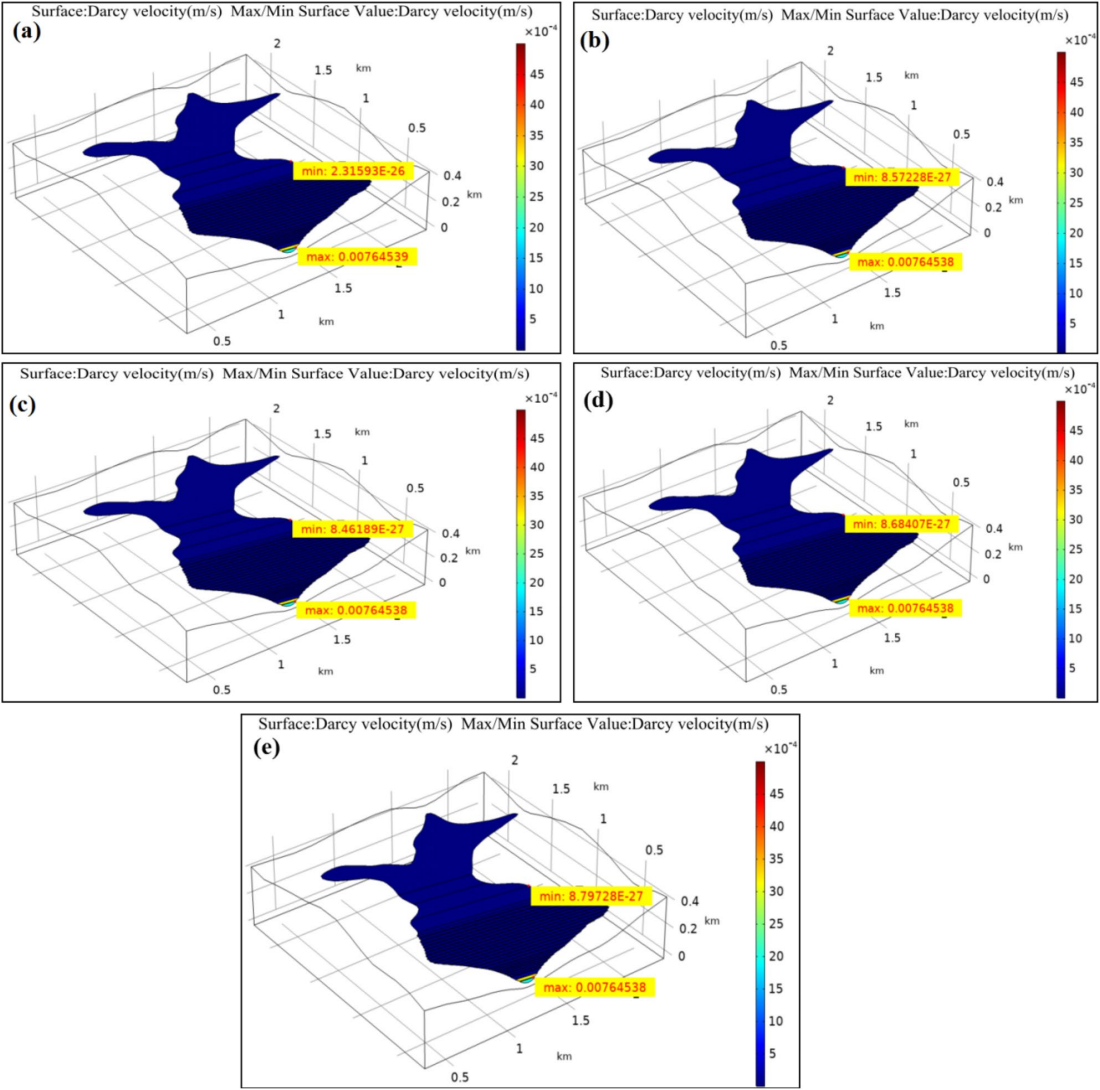
Velocity field distribution of the tailings dam under different pH values conditions. (**a**) pH = 7 (original sample); (**b**) pH = 3; (**c**) pH = 5; (**d**) pH = 9; (**e**) pH = 12.

Chemical solution will develop a series of physicochemical reactions with the tailings sedimentary soil, which will thereby result in dissolution or precipitation and crystallization to varying degrees^32–34^. As a result, the permeability coefficient of tailings is also affected, resulting in the different fluid flow rates in the dam. The velocity field distribution inside the dam under the treatment of chemical solutions with different pH values was obtained according to the analysis on the dam velocity field, as shown in Fig. 3. In Fig. 3, the changes in maximum flow rate and minimum flow rate in Darcys velocity field under various working conditions with pH value were not the constant values. The maximum and minimum values of Darcys velocity under different working conditions are summarized in Table 3.

**Table 3:** Values of Darcys velocity under different conditions.

Conditions	Maximum values *cm*/*s*	Minimum value *cm/s*
1: pH = 7 (Original sample)	7.64539 × 10^–1^	2.31593 × 10^–24^
2: pH = 3	7.64538 × 10^–1^	8.57228 × 10^–25^
3: pH = 5	7.64538 × 10^–1^	8.46189 × 10^–25^
4: pH = 9	7.64538 × 10^–1^	8.68407 × 10^–25^
5: pH = 12	7.64538 × 10^–1^	8.79728 × 10^–25^

**Figure 4:**
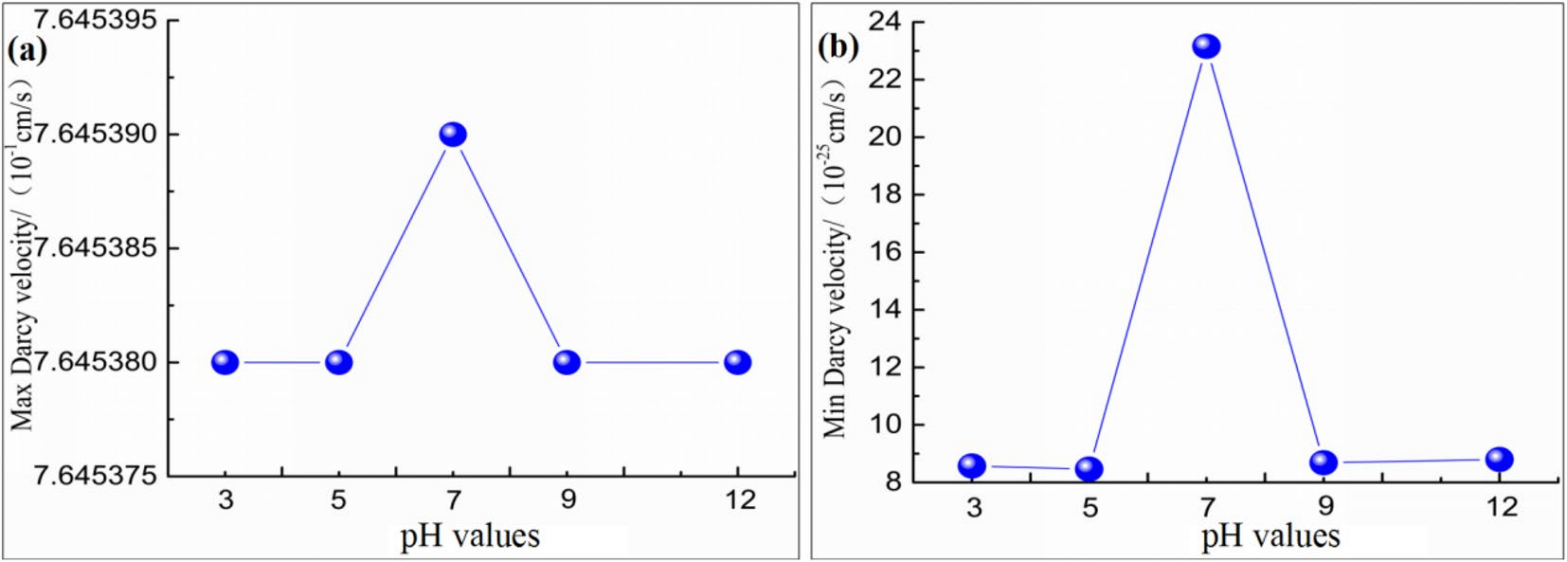
Changes in Darcys velocity with with the solution pH values. (**a**) Maximum velocity; (**b**) Minimum velocity.

According to Table 3, the variation diagram of maximum and minimum values of Darcys velocity as a function of pH was plotted in Fig. 4, where pH = 7 represents the original sample working condition.

It was illustrated from Fig. 4 that, from working condition 1 to working condition 5, the maximum and minimal values of fluid flow velocity inside the tailings dam showed an overall decreasing trend with the changes in reservoir water acidity and alkalinity, which was partially related to the changes in tailings material porosity. In the meantime, the maximum Darcys velocity occurred near the initial dam, and such phenomenon was linked with the permeability of initial dam, therefore, the maximum velocity was not obviously changed. The minimum Darcys velocity occurred inside the dam; under original sample condition, the minimum Darcy velocity inside the dam was about 2.72 folds of that in acid environment and 2.65 folds of that in alkaline environment.

The possible reason was that, acid solution damaged the tailings materials, which resulted in tailings particle refining, decreased permeability coefficient, and continuously reduced flow rate in the dam. In addition, the alkaline solution had corrosion and cementation effects on the tailings materials, which also reduced the permeability coefficient and led to the continuously decreased flow rate inside the dam^33–35^. Thus, it is obvious that, both acid and alkaline reservoir water deteriorates the permeability of tailings materials and reduces the permeability velocity in the dam, which is to the disadvantage of seepage drainage.

#### Analysis of effective saturation

**Figure 5:**
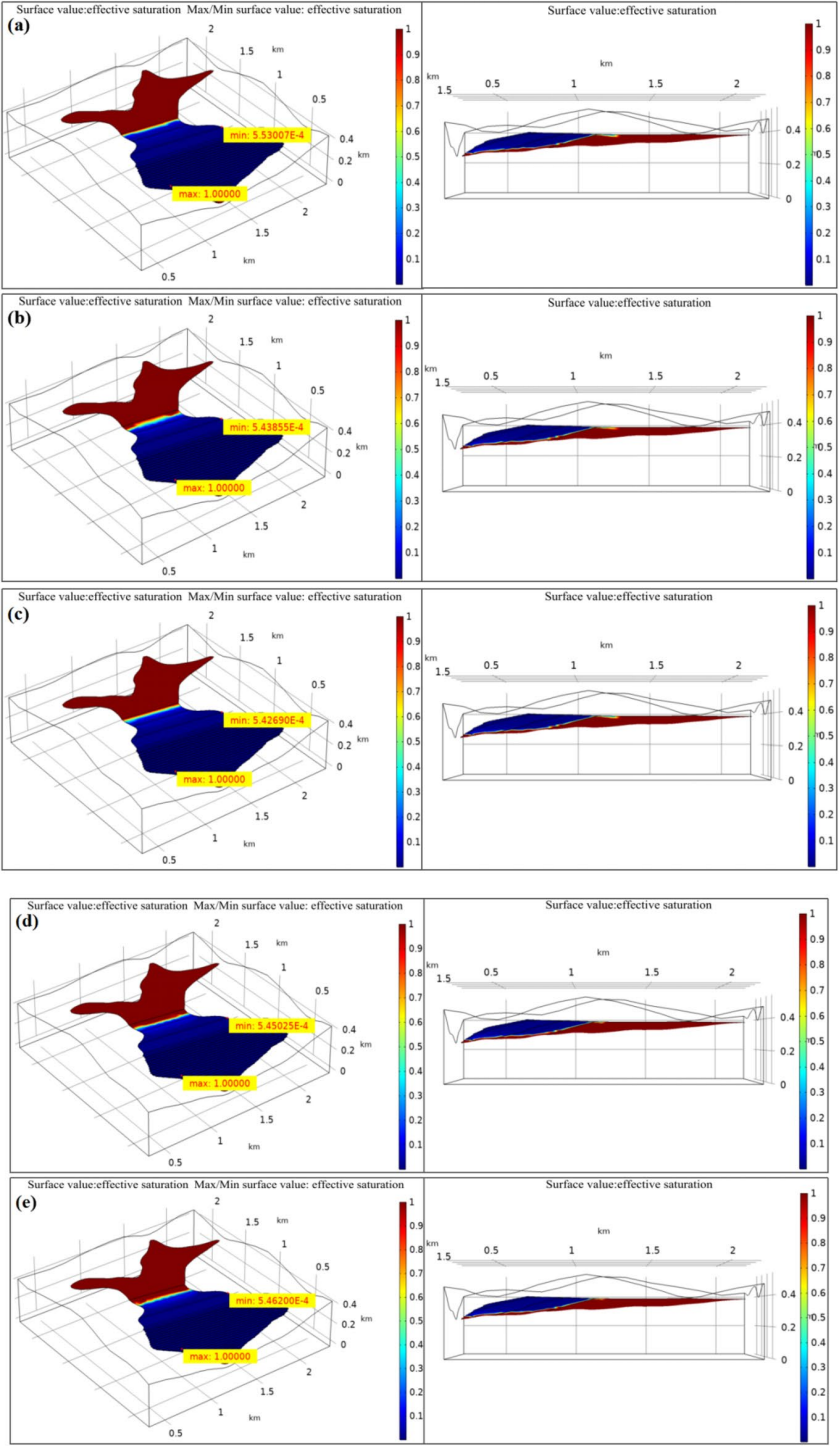
Effective saturation of the tailings dam under different pH values conditions. (**a**) pH = 7 (original sample); (**b**) pH = 3; (**c**) pH = 5; (**d**) pH = 9; (**e**) pH = 12.

Figure 5 shows the simulation results of dam effective saturation under different permeability coefficients. It was obvious that, both the effective saturation and dry beach length of tailings reservoir changed with the change in permeability coefficient, as shown in Table 4. Under the five working conditions, the maximum and minimum saturation values were not greatly changed, among which, the maximum effective saturation was measured at the dam base, with the value of 1. It indicated that the tailings was completely saturated, while the minimum effective saturation was detected near the dry beach surface of the fill dam crest, with the neglectable value of as low as 10^–5^ ~ 10^–6^. It suggested that the changes in permeability coefficient had little influence on the maximum or minimum effective saturation. In addition, it was also discovered based on the changes in dry beach length of tailings reservoir that, the dry beach length exhibited a significantly decreasing trend with the acidity-alkalinity of reservoir water.

**Table 4:** Effective saturation and dry beach length of tailings reservoir under different pH values conditions.

Conditions	Maximum effective saturation	Minimum effective saturation	Dry beach length (*m*)
1: pH = 7 (Original sample)	1	5.53007 × 10^–4^	447
2: pH = 3	1	5.43855 × 10^–4^	348
3: pH = 5	1	5.42690 × 10^–4^	417
4: pH = 9	1	5.45025 × 10^–4^	298
5: pH = 12	1	5.46200 × 10^–4^	249

**Figure 6:**
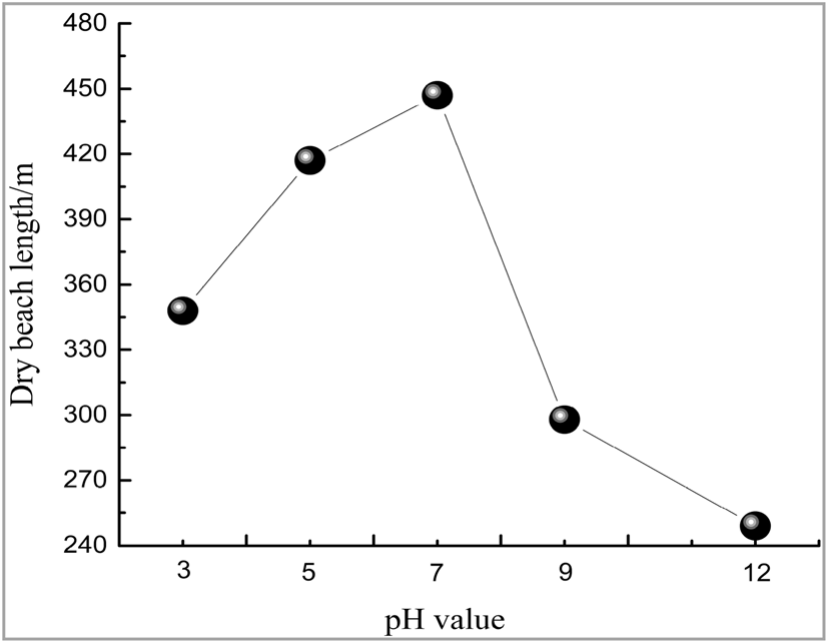
Variation trend line of dam dry beach length with pH values.

Figure 6 shows the variation trend line of dam dry beach length with pH values. As observed, the tailings dam dry beach length showed decreasing trends under both acid and alkaline environment, and the stronger acidity-alkalinity of reservoir water led to shorter dry beach length. On the whole, the decreasing amplitude of dry beach length under alkaline environment was greater than that under acid environment. Combined with the test results, changes in environmental acidity-alkalinity led to changes in dam permeability, besides, tailings materials suffered from a greater decrease in permeability coefficient in alkaline solution than in acid solution, thus slowing down the fluid flow rate inside the tailings dam and leading to choking tailings seepage drainage. From the macroscopic perspective, the overall effective saturation of tailings dam increased, the phreatic line elevated, the dry beach face shortened, and more obvious changes were observed under alkaline environment.

In this analysis, the infiltration point of tailings seepage field was located at the initial dam rather than the fill dam, and the dry beach length was greater than the minimum dry beach length stipulated in the tailings design specifications. However, if the tailings dam is in the acid-alkaline environment for a long time during the use process, it will still lead to continuously elevated tailings reservoir water level and persistently shortened dry beach length, finally giving rise to dam failure under the seepage action^36,37^. Therefore, the acid-alkaline environment greatly affects the dam stability.

### Influence of tailings permeability coefficient ratio on dam phreatic line

In this study, the tailings dam section along the valley bottom was selected as the basic section for calculation, and the influences of tailings permeability coefficient ratios between each layer on the dam phreatic line were investigated using the GeoStudio software (2007 version, Calgary, Alberta, Canada)^38–40^. The soil structure was basically abstracted into three layers, namely, the tailings silty sand, tailings silty soil and tailings silty clay. Then, based on the constructed model, two programs including eight working conditions were set, as shown in Table 5.

**Table 5:** Tailings permeability coefficient ratios between each layer.

Program 1	Permeability coefficient ratio $k_{a}$: $k_{b}$: $k_{c}$:$k_{d}$	Program 2	Permeability coefficient ratio $k_{a}$: $k_{b}$: $k_{c}$:$k_{d}$
Condition 1	1: 100: 1: 50,000	Condition 5	500: 100: 1: 50,000
Condition 2	10: 100: 1: 50,000	Condition 6	1000: 100: 1: 50,000
Condition 3	50: 100: 1: 50,000	Condition 7	5000: 100: 1: 50,000
Condition 4	100: 100: 1: 50,000	Condition 8	10,000: 100: 1: 50,000

#### Dam phreatic line distribution

**Figure 7:**
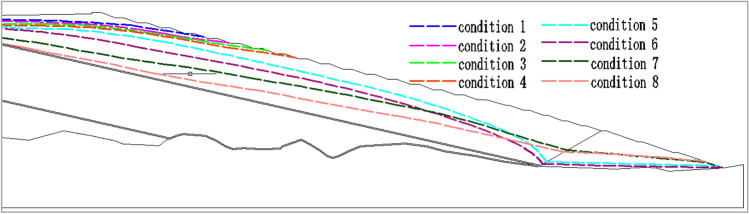
Dam phreatic line distribution under different conditions.

The dam phreatic line is the intersecting line between the seepage flow surface and the dam body cross section, which is the direct manifestation of tailings dam stability. Figure 7 exhibits the dam phreatic line distribution in each layer under different permeability coefficient ratios. In program 1, the phreatic line elevation showed an extension trend from downstream to upstream in the case of extremely great differences in the permeability coefficient of tailings material among various layers. In program 2, when the ratio of upper layer permeability coefficient to the lower layer in the tailings dam increased, the phreatic line in the downstream dam gradually elevated; moreover, with the increase in ratio, the phreatic line increasingly elevated, the height of infiltration point also increased obviously, and the buried depth of phreatic line became shallow. In addition, the drop phenomenon was obvious when the permeability coefficient was small, which gradually became weakened and even disappeared with the increase in permeability coefficient ratio.

#### Analysis of infiltration point height of phreatic line

The infiltration point height of dam phreatic line under different working conditions was slightly different, as presented in Table 6.

**Table 6:** Infiltration point height of dam phreatic line under different conditions.

Program 1	Infiltration point height/*m*	Program 2	Infiltration point height/*m*
Condition 1	122	Condition 5	1.73
Condition 2	121	Condition 6	0.48
Condition 3	110	Condition 7	2.66
Condition 4	99	Condition 8	5.67

**Figure 8:**
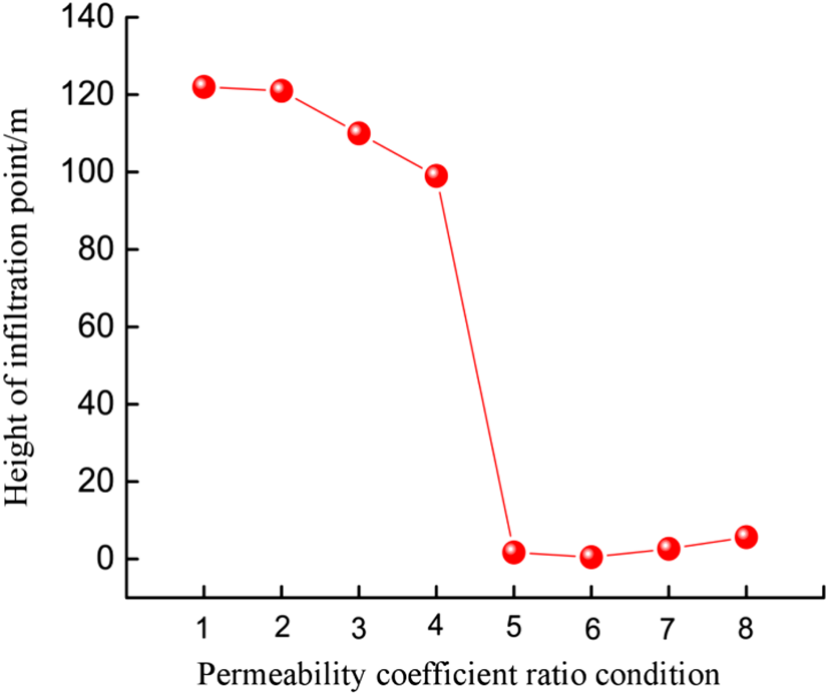
Variation trend for the infiltration point height of dam phreatic line under different permeability coefficient ratio conditions.

Figure 8 is the variation trend chart for the infiltration point height of phreatic line under different permeability coefficient ratios. As observed from the figure, the infiltration point height in program 1 was far greater than that of program 2. In program 1, when the permeability coefficient of the first layer was lower than the second layer, the infiltration point was high, along with great variation amplitude (as high as 23 m). In program 2, the infiltration point was low when the permeability coefficient of the first layer was greater than the second layer, and the infiltration point height showed little changes among the four working conditions in program 2, with the maximum of only 5.19 m. The variation of infiltration point height in program 1 was 4.4 folds of that in program 2. Under two conditions, the infiltration point of phreatic line elevated as the permeability coefficient ratio of the upper layer to the lower layer of tailings dam increased, and these two showed positive correlation. Based on the actual situation of the tailings reservoir, the infiltration points under the four working conditions in program 1 were at the fill dam, which easily led to seepage failure and was to the disadvantage of dam stability. In program 2, the infiltration points were at the initial dam, and tailings dam at such condition was more stable^15,41^.

#### Buried depth of dam phreatic line

**Figure 9:**
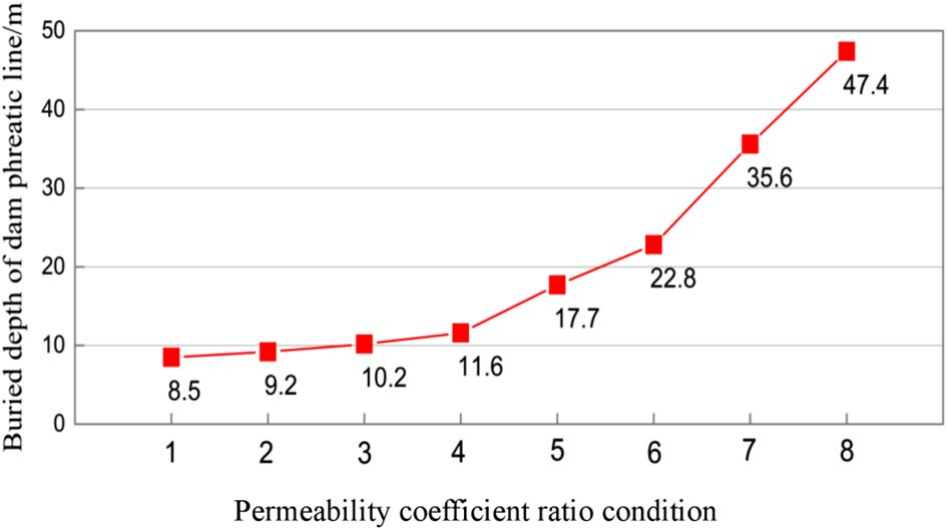
Variation trend for the buried depth of dam phreatic line under different permeability coefficient ratio conditions.

The buried depth of phreatic line at the direction perpendicular to the fill dam crest was selected as the analysis data (Table 7). Figure 9 shows the influence of permeability coefficient ratio on the buried depth of phreatic line. Clearly, the variations of phreatic line buried depth with permeability coefficient ratio were opposite under two conditions.

**Table 7:** Buried depth of dam phreatic line under different conditions.

Program 1	Buried depth of dam phreatic line/*m*	Program 2	Buried depth of dam phreatic line/*m*
Condition 1	8.5	Condition 5	17.7
Condition 2	9.2	Condition 6	22.8
Condition 3	10.2	Condition 7	35.6
Condition 4	11.6	Condition 8	47.4

In program 1, the buried depth of phreatic line gradually became deeper with the decrease in permeability coefficient, but the overall variation amplitude of phreatic line buried depth was small, with the maximum difference in phreatic line buried depth of 3.1 m. When the permeability coefficient of the first layer was smaller than or equal to that of the second layer, the phreatic line buried depth of tailings dam gradually increased with the decrease in permeability coefficient ratio, and there was inverse relation between the two. In program 2, the overall phreatic line became deepened with the increased in permeability coefficient of each layer in the dam, and obvious variation was observed, with the maximum difference of 29.7 m. When the permeability coefficient of the first layer was greater than that of the second layer and when the permeability coefficient ratio increased, the phreatic line buried depth of tailings dam gradually increased, and there was proportional relation between the two. However, on the whole, the phreatic line buried depth of tailings dam was positively correlated with the permeability coefficient of the first layer.

To sum up, to increase the phreatic line buried depth and enhance the dam stability, the permeability coefficient of the upper layer tailings material should be greater than that of the lower layer tailings material.

## Conclusions

Based on the actual engineering, this study carries out permeability tests to analyze the influences of acidity-alkalinity on the permeability characteristics of tailings materials. It also investigates the effects of multi-factorial conditions on the dam seepage field on the basis of numerical simulation. The following conclusions are drawn.

After chemical solution treatment, the permeability coefficient of tailing materials shows a decreasing trend compared with that in original tailings samples; with the increase in immersion time, the reaction between the chemical solution and the tailings materials becomes more sufficient, which exerts a more obvious clogging effect on the tailings materials, leading to the decreased porosity of tailings materials and reduced permeability coefficient.

When the reservoir water gradually becomes acid or alkaline, the maximum and minimum fluid flow rates in the tailings dam decrease on the whole. In addition, the dry beach length of tailings dam also shows a decreasing trend, and the dry beach length is shorter as the reservoir water acidity-alkalinity becomes stronger. On the whole, the decreasing amplitude of dry beach length in alkaline environment is greater than that in acid environment.

In general, the infiltration point of the dam phreatic line increases with the increase in the permeability coefficient ratio of the upper layer to the lower layer. Moreover, there is positive correlation between the phreatic line buried depth of the tailings dam and the upper layer permeability coefficient. The increase in upper layer permeability coefficient results in the gradually deepened phreatic line buried depth of the tailings dam.
